# Revealing the planar chemistry of two-dimensional heterostructures at the atomic level

**DOI:** 10.1038/ncomms8482

**Published:** 2015-06-23

**Authors:** Harry Chou, Ariel Ismach, Rudresh Ghosh, Rodney S. Ruoff, Andrei Dolocan

**Affiliations:** 1 Department of Mechanical Engineering, The University of Texas at Austin, Austin, Texas 78712, USA; 2 Microelectronics Research Center, The University of Texas at Austin, Austin, Texas 78758, USA; 3 Department of Materials Science and Engineering, Tel Aviv University, Ramat Aviv 6997801, Israel; 4 Center for Multidimensional Carbon Materials, Institute of Basic Sciences Center, Ulsan National Institute of Science and Technology, Ulsan 689-798, Republic of Korea; 5 Texas Materials Institute, The University of Texas at Austin, Austin, Texas 78712, USA

## Abstract

Two-dimensional (2D) atomic crystals and their heterostructures are an intense area of study owing to their unique properties that result from structural planar confinement. Intrinsically, the performance of a planar vertical device is linked to the quality of its 2D components and their interfaces, therefore requiring characterization tools that can reveal both its planar chemistry and morphology. Here, we propose a characterization methodology combining (micro-) Raman spectroscopy, atomic force microscopy and time-of-flight secondary ion mass spectrometry to provide structural information, morphology and planar chemical composition at virtually the atomic level, aimed specifically at studying 2D vertical heterostructures. As an example system, a graphene-on-h-BN heterostructure is analysed to reveal, with an unprecedented level of detail, the subtle chemistry and interactions within its layer structure that can be assigned to specific fabrication steps. Such detailed chemical information is of crucial importance for the complete integration of 2D heterostructures into functional devices.

## Abstract (web summary)

The properties of 2D materials such as graphene can vary according to the quality and, for vertical devices, the interfaces between materials. Here, the authors report a method using TOF-SIMS, micro-Raman spectroscopy and atomic force microscopy to give high levels of detail of vertical 2D heterostructures.

## Graphical abstract

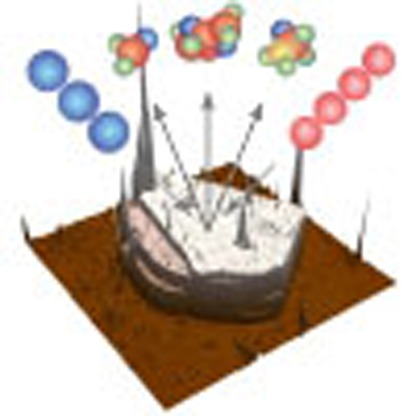

Since the experimental isolation of graphene^1^, there has been a renewed interest in two-dimensional (2D) atomic crystals and their heterostructures in the context of their potential for various types of electronic devices, among other applications^2^,^3^,^4^,^5^,^6^. Including single to few-layer graphene, hexagonal boron nitride (h-BN) and metal dichalcogenides (for example, MX_2_ where M=Mo, Hf, W, Nb and so on and X=S, Se or Te), 2D materials exhibit unique and synergistic properties^2^,^5^,^6^. Combining such 2D materials to fabricate ultra-thin vertical heterostructures might enable advanced nanodevices with outstanding capabilities^6^,^7^. One key step in the fabrication sequence of a 2D heterostructure is the understanding of its material properties at the atomic level, which allows for fundamental improvements in manufacturing advanced nanodevices. As the vertical heterostructures consisting of 2D systems contain atomically thin interfaces, analytical techniques that reveal the planar chemical composition of buried interfaces with atomic resolution are needed. To date, all common fabrication methods of 2D heterostructures, including mechanical exfoliation^8^,^9^ and polymer-based lift-off procedures^8^,^10^, introduce contamination as the 2D materials are exposed to several environments before the final stacking configuration. Given that the ‘quality' of a 2D heterostructure is directly linked to the amount, location and composition of the contained contamination, characterization and mitigation of residues (contamination) are the subject of ongoing research^6^. Raman spectroscopy^11^,^12^, electrical characterization^13^, X-ray photoelectron spectroscopy (XPS) and transmission electron microscopy (TEM) are commonly used to determine the ‘quality' of 2D materials following the transfer and subsequent treatment processes. In the case of graphene, the energy shift of its Raman peaks is a measure of doping^12^,^14^, whereas XPS and TEM can detect polymer and other residues by either identifying specific bonds in the polymer(s)^12^ or by direct visualization^15^. To date, all known surface characterization techniques have limitations when dealing with low doping or contamination concentrations in ultra-thin (less than few nanometres) films, mostly related to their intrinsic in-depth chemical selectivity and sensitivity. To name a few, purely from the elemental and surface sensitivity perspective, besides requiring a >0.1% elemental concentration for detection, Auger electron spectroscopy, XPS, Raman and energy-dispersive X-ray spectroscopy output the averaged chemical information from a depth varying from at least 1 nm to a few hundreds nm (the escape depth of the analysed particles). Possessing ultra-high (virtually atomic) in-depth chemical selectivity and parts-per-billion sensitivity, time-of-flight secondary ion mass spectrometry (TOF-SIMS) is a good candidate for characterizing the composition of 2D heterostructures. As an example, in conjunction with atomic force microscopy (AFM), TOF-SIMS can accurately provide fundamental features such as atomic mixing and chemical composition at buried interfaces, which are commonly unavailable by applying TEM or scanning electron microscopy with energy-dispersive X-ray spectroscopy on cross-sectional samples^16^. Previously, TOF-SIMS depth profiling and high-resolution imaging have been used to map individual layers in isotope-labelled multilayer graphene^17^.

Here, TOF-SIMS is applied, for the first time to our knowledge, to a 2D atomic crystal heterostructure fabricated by common sequential layer transfer, to study its planar chemical composition at the atomic level, with significant implications for understanding the influence of manufacturing and transfer processes. As a system of choice, the conductor-on-insulator stacking of graphene on h-BN shows promise as a base for high-quality nanoelectronics^11^,^18^. Sharing a nearly identical structure with graphene^19^, monolayer h-BN is known to be electronically ‘decoupled' from graphene, which results in graphene having significantly higher electrical conductivity when compared with other insulating substrates, such as silicon dioxide^13^,^18^. For instance, studies showed that an h-BN/graphene/h-BN structure maintains pristine graphene properties and protects it from environmental changes, such as temperature stress^11^. In general, the properties of graphene depend on the choice of substrate in its final state and the environments in contact with its surfaces during preparation. In particular, the graphene-on-h-BN heterostructure shows contamination due to the chemical vapour deposition (CVD) of graphene, its solvent lift-off from the growth substrate (that is, copper foil) and deposition onto the new substrate (that is, h-BN flakes on SiO_2_). Our characterization approach is able to detect, identify, localize and quantify the various contaminants introduced during fabrication, thus leading to improved manufacturing processes and, therefore, better-performing devices.

## Results

### General characterization of the transferred graphene

**Figure 1:**
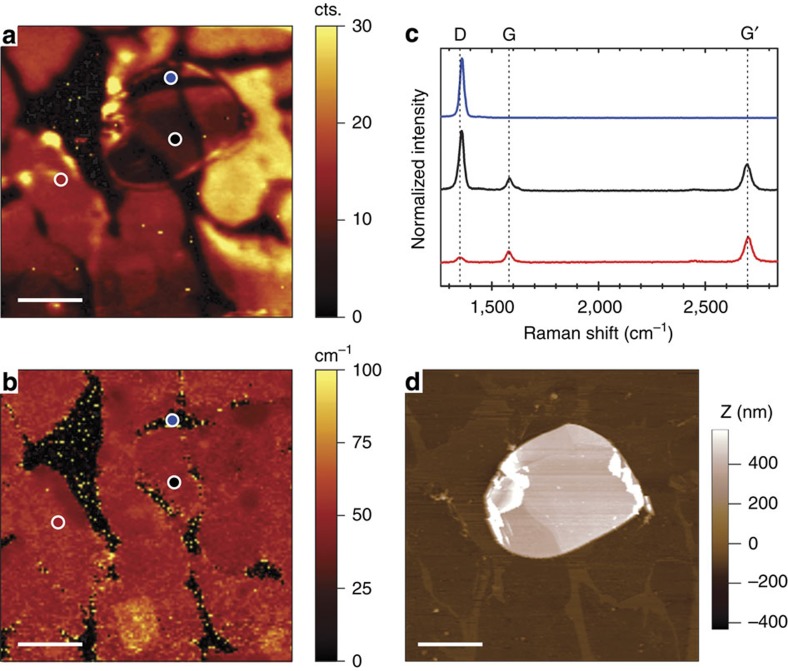
Micro-Raman and AFM characterization of the transferred graphene. (**a**–**c**) Raman and (**d**) AFM characterization of the 2D heterostructure consisting of a CVD-grown, wet-transferred graphene on an h-BN flake supported by SiO_2_. G′ Raman peak intensity (**a**) and FWHM (**b**) map of the same location. (**c**) Raman spectra at the locations indicated in **a** and **b**, where the black and red spectra correspond to single-layer graphene atop h-BN and SiO_2_, respectively, whereas the blue spectrum represents the h-BN flake exposed between adjacent graphene grains. The raw Raman intensities for the blue and black regions were normalized to their respective E_2g_ h-BN peak maximum, whereas for the red region, the G′ band maximum was matched to the corresponding one in the black region. (**d**) AFM height topography of graphene patches transferred on top of either an h-BN flake or the surrounding SiO_2_ substrate. Scale bars, 10 μm.

To evaluate its final configuration, the 2D system, composed of a CVD-grown, wet-transferred graphene onto a SiO_2_ substrate that was partially covered by mechanically exfoliated h-BN flakes, was first characterized by (micro-) Raman spectroscopy (Fig. 1a–c) and AFM (Fig. 1d). Initially supported by a copper foil, the CVD-grown graphene did not fully cover its substrate, rendering domains between 10 and 20 μm across^20^. After the wet transfer process, both micro-Raman mapping of the graphene overtone G′ (or 2D) band^21^ around ∼2,700 cm^−1^ and AFM height topography confirmed large (tens of microns), continuous graphene patches extending over the h-BN flakes and SiO_2_ substrate (Fig. 1a,d, respectively). Usually associated with the number of stacked graphene layers, the G′ peak full width at half maximum (FWHM) remains roughly constant at ∼30 cm^−1^ (Fig. 1b) over large areas, suggesting that the monolayer structure is preserved after transfer^21^,^22^. In addition, Raman spectra acquired at representative locations indicated in Fig. 1a,b are shown in Fig. 1c, where the red and black curves, corresponding to the transferred graphene supported by SiO_2_ and h-BN, respectively, exhibit a G to G′ peak ratio of <0.5 thus implying single-layer graphene domains^23^. Atop the h-BN flake, in between the graphene domains, the E_2g_ vibrational mode associated with in-plane transversal and longitudinal optical phonons of the bulk h-BN structure can be observed. Located around 1,365 cm^−1^ in the Raman spectrum (Fig. 1c, blue curve), this peak is far more intense than the defect-induced D peak for graphene at ∼1,350 cm^−1^. Therefore, in the graphene-coated h-BN region, the D band is not easily detected by Raman spectroscopy (at the laser wavelength used in this study, that is, 488 nm). However, atop SiO_2_ the graphene D peak is disentangled from any substrate-related signal and presents a relatively low intensity compared with the G peak (that is, D- to G-peak intensity ratio of about 0.4), thus confirming a relatively small defect density of the graphene domains (that is, it relates to the domain boundaries rather than its structural consistency).

### TOF-SIMS/AFM-combined investigation of the 2D system

**Figure 2:**
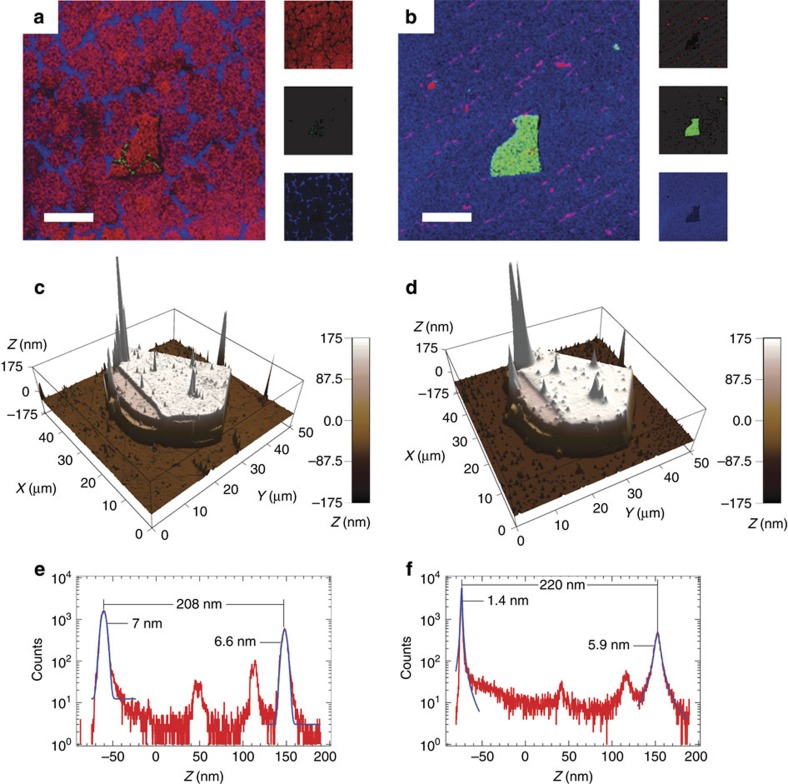
TOF-SIMS/AFM as an alternate characterization method for the transferred graphene. TOF-SIMS secondary ion maps acquired in high lateral resolution (burst alignment mode with no bursts; Bi_1_^+^ at 30 keV ion energy) overlaying three species of interest C^−^ (red), B^−^ (green) and O^−^ (blue), which correspond to graphene, h-BN and SiO_2_, respectively. (**a**) The as-deposited transferred graphene system shows patches that extend over the h-BN flake. (**b**) Sixty seconds of Cs^+^ (500 eV ion energy) sputtering completely removes the graphene overlayer revealing the h-BN flake. Scale bars, 20 μm. (**c**,**d**) AFM height topography of an h-BN flake covered by graphene before and after 35 s of Cs^+^ (500 eV ion energy) sputtering. (**e**,**f**) Height distributions (that is, histograms) of the AFM maps in **c** and **d**, respectively. Sputtering induces a strong reduction of surface corrugation as inferred by the change in shape and width of the two main histogram features. The h-BN flake investigated in **a** and **b** is different from the flake investigated in **c**–**f**, and the flake investigated in Fig. 1.

Figure 2 shows TOF-SIMS high lateral resolution (∼200 nm) maps of three species of interest, C^−^, B^−^ and O^−^, representing graphene, h-BN and the SiO_2_ substrate, respectively, before (Fig. 2a) and after (Fig. 2b) ∼60 s of Cs^+^ (500 eV energy) sputtering. As deposited (Fig. 2a), the transferred graphene exhibits large, tens of microns across, domains that span both the SiO_2_ substrate and the h-BN flake, confirming the AFM and Raman results. Represented by the O^−^ signal, the exposed SiO_2_ substrate indicates the boundaries of these graphene patches, some likely crumpled as a consequence of the transfer process^23^. After ∼60 s of Cs^+^ sputtering, the graphene overlayer is completely removed revealing the h-BN flake location and traces of organic residues at the SiO_2_ surface. As a result, besides the high lateral resolution (∼200 nm) that is better or comparable to micro-Raman (Fig. 1a,b), TOF-SIMS adds the fundamental ability to ‘chemically separate' the graphene overlayer from the substrate. Consequently, by controlling the removal of the outer layers at a very slow rate (typically <0.1 nm s^−1^), we can progressively study the outer adsorbed species, the top graphene layer, the interfacial contaminants and the underlying h-BN and SiO_2_.

The secondary ions collected during depth profiling are subject to the morphology and the electronic properties of the surface from which they are sputtered. In particular, the initial roughness and any roughness induced through sputtering will affect the depth resolution of collected profiles^24^. To (i) understand the physical effects of sputtering on the graphene/h-BN and graphene/SiO_2_ interfaces and (ii) estimate sputtering rates for the species of interest, the surface topography of the 2D heterostructure was investigated by AFM before (Fig. 2c) and after (Fig. 2d) 35 s of sputtering with Cs^+^ at 500 eV energy. Both height distributions (histograms) shown in Fig. 2e,f, corresponding to the height topographies in Fig. 2c,d, respectively, exhibit two main peaks attributed to the SiO_2_ substrate (*Z*≈−65 nm) and h-BN flake surface (*Z*≈150 nm). An obvious change in shape from Gaussian to Lorentzian and a significant reduction of the width (FWHM) for the two main histogram peaks suggest that Cs^+^ sputtering substantially decreases the surface corrugation of both the SiO_2_ substrate and h-BN flake surface (for more details see Supplementary Figs 1 and 2 and Supplementary Note 1). In addition, the corrugation at an interface is considered to be an average of the corrugations measured before and after sputtering through that interface^16^,^25^.

### Atomic mixing between graphene and substrates

**Figure 3:**
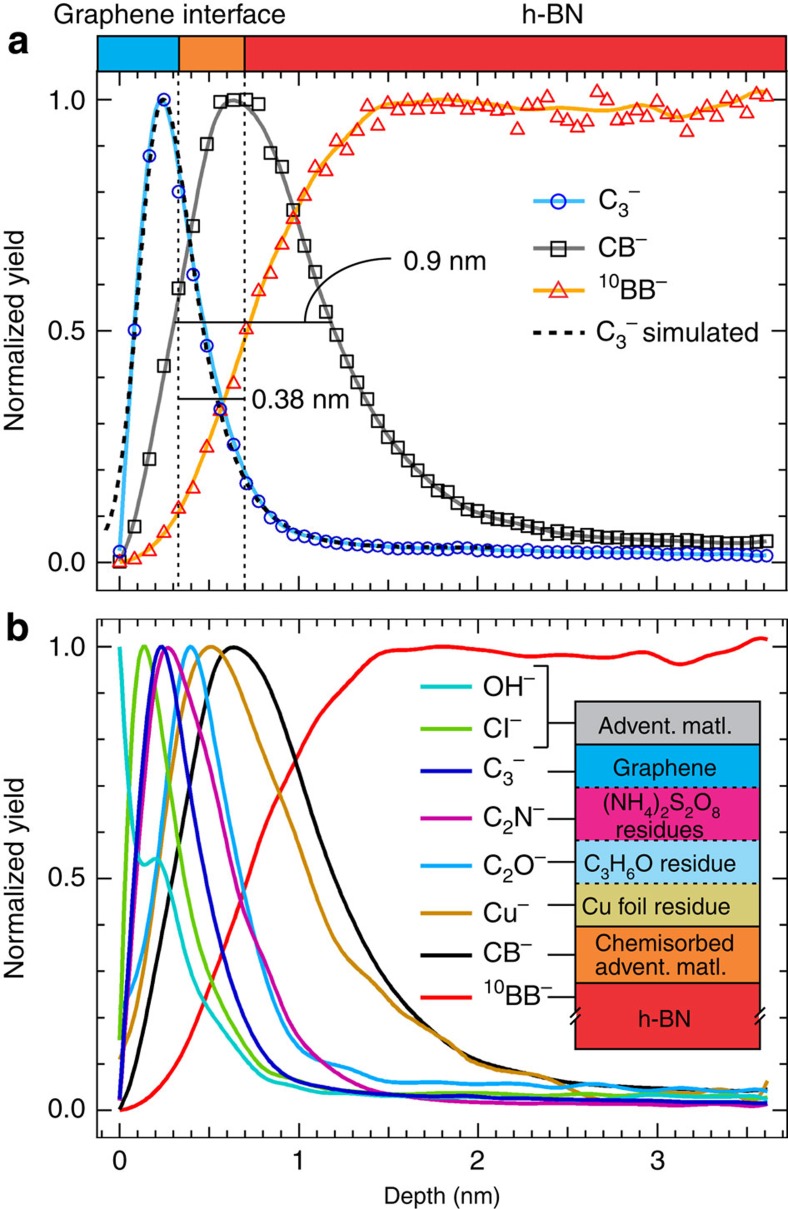
Vertical composition of the transferred graphene/h-BN system. (**a**) Normalized (to maximum) TOF-SIMS depth profiles of C_3_^−^, CB^−^ and ^10^BB^−^ representing single-layer graphene, adventitious organic material chemisorbed at the h-BN surface and the h-BN substrate, respectively. The black dashed line represents the simulated C_3_^−^ profile as described in the text. (**b**) Additional species of interest corresponding to surface adsorbates (Cl^−^ and OH^−^) and interfacial contaminants from the transfer process (C_2_N^−^, C_2_O^−^ and Cu^−^). The discrete markers represent the real data while the continuous curves represent the 1-point spline interpolations. The h-BN flake investigated here is different from the h-BN flakes investigated in Figs 1 and 2. Analysis conditions: analysis ion beam: Bi_1_^+^ (high-current mode, 30 keV ion energy, ∼3 pA sample current, probing area of 100 × 100 μm^2^); sputtering ion beam: Cs^+^ (500 eV ion energy, ∼45 nA sample current, sputtering area of 200 × 200 μm^2^).

In Fig. 3, TOF-SIMS depth profiling was employed to reveal the vertical chemical composition of the 2D heterostructure. The sputtering time was converted into depth by applying a sputtering-rate model assuming the instantaneous sputtering rate at the graphene/h-BN interface as a linear combination of the individual sputtering rates^16^,^25^ (Supplementary Fig. 3; Supplementary Note 2). Figure 3a shows the depth profiles of C_3_^−^, CB^−^ and ^10^BB^−^ secondary ions (normalized to maximum), both the acquired data (discrete markers) and their 1-point spline interpolations (continuous curves), representing single-layer graphene, adventitious organic material chemisorbed at the h-BN surface and the h-BN flake, respectively. Owing to the discontinuous nature of the transferred graphene (tens-of-microns-sized patches), the depth profiles on both the graphene/h-BN and graphene/SiO_2_ systems were reconstructed from the initial data using regions of interest that ensured graphene coverage (that is, coverage of the C_3_^−^ species). For depth profiling, polyatomic species such as C_3_^−^ and ^10^BB^−^ were chosen to avoid the intrinsic artefacts that monoatomic species such as C^−^ and B^−^ have due to residuals from oxides or other chemisorbed adventitious species. An estimation of the atomic mixing length between graphene and h-BN or SiO_2_ can be obtained by applying the so-called mixing–roughness–information (MRI) model^26^ to the measured interface thickness (more details in the Supplementary Note 3). For a given interface between two materials, this model is based on three major assumptions: (1) the real interface (equivalent with the intrinsic atomic mixing between the two materials following fabrication) will appear broadened upon depth profiling due to three phenomenological factors: (a) sputtering-induced atomic mixing, (b) intrinsic and sputtering-induced corrugation and (c) actual signal depth of origin (equivalent with the escape depth of the analysed secondary ions) at the regressing surface; (2) these three factors can be disentangled and considered as independent from each other and (3) they can be described by analytical functions of depth whose convolution defines the so-called depth resolution function. Consequently, by deconvoluting the depth resolution function from the measured interface thickness obtained by depth profiling, one can extract the real interface thickness (that is, the fabrication-induced (or real) atomic mixing length, Supplementary Fig. 3a). At this stage, we emphasize a note of caution: atomic mixing and roughness cannot be, in reality, completely disentangled. Within the MRI model, however, these two quantities are considered to be independent, thus one must clearly define the roughness such that it is completely separated from atomic mixing. In this case, we define the roughness as the root-mean-square roughness given by a scanning probe microscopy (SPM) tool, for example, AFM. To simulate the C_3_^−^ profile (normalized to its maximum), we use the forward calculation procedure^26^ where we assume some starting values for the real atomic mixing, sputtering-induced mixing, roughness and information parameters and convolute the associated functions to obtain the simulated profile, which is then fitted to the actual C_3_^−^ data points. The fitting procedure searches for the minimum deviation between the simulated profile and the C_3_^−^ profile points (normalized to maximum and stretched to 0, see Supplementary Fig. 4 and Supplementary Note 3) while varying all parameters in steps of ±0.01 nm from their starting values. In the case of the graphene/h-BN interface, the simulated C_3_^−^ profile is appended in Fig. 3a as the black dashed curve and shows good agreement with the data. Following the simulation, the real atomic mixing length between graphene and h-BN is found to be negligible (<0.01 nm). Hence, the measured interface thickness extracted from depth profiling of the graphene/h-BN system is generated by corrugation and minimal sputtering effects. Thus, the graphene overlayer proves to be chemically inert with respect to the h-BN substrate. Additional evidence of non-mixing is given by the far smaller measured C_3_^−^ interface thickness, ∼0.38 nm, than the FWHM of the mixed graphene/h-BN species, CB^−^, ∼0.9 nm, the latter being a result of the chemisorbed organic species at the h-BN surface before graphene transfer (Fig. 3; Supplementary Fig. 5; Supplementary Note 4). In contrast, the graphene/SiO_2_ system presents a measured interface thickness of ∼0.87 nm (Supplementary Fig. 6a), which is comparable to the FWHM of the SiC^−^ profile, ∼1.1 nm. In this case, the application of the forward calculation for the C_3_^−^ profile in the MRI model yields a nonzero real atomic mixing length (∼0.2 nm). This is a result of the chemisorbed species (identified below) accumulated in graphene during the transfer process and which are chemically interacting with the SiO_2_ substrate (Supplementary Fig. 6; Supplementary Note 4). Physisorbed vs chemisorbed species are discussed in Supplementary Note 5.

### Identification, localization and quantification of residues

Additional residuals from the transfer process at the graphene/h-BN interface include S^−^, C_2_N^−^, C_2_O^−^ and Cu^−^ species. Albeit in small amounts, S^−^ and C_2_N^−^ species show the same depth profile localization as C_3_^−^, thus indicating a chemical interaction between graphene and the lift-off solvent, (NH_4_)_2_S_2_O_8_ (Supplementary Fig. 7a). An oxidized, organic, partial monolayer (∼0.4 nm thick), presumably an acetone or, most probably, a poly(methyl methacrylate) (PMMA)/acetone residue and represented by the C_2_O^−^ marker, can be observed right under the graphene, followed by a third layer containing traces of copper residue. In fact, a closer look at the shape of the C_2_N^−^ depth profile suggests that most of its signal originates from three atomic-like layers, confirming the three-layered structure of the transferred graphene system (Supplementary Fig. 8). The corresponding fit of the C_2_N^−^ profile leads to a simple coverage approximation of these three layers at 1, 0.58 and 0.19 monolayer, respectively, where the first layer is considered to be, roughly, a full monolayer graphene, whereas the second and third are a combination of organic residuals consisting of nitriles, carbon sulfides and organic oxides that result from the copper wet-etching and PMMA removal procedures. Finally, the amount of copper in the transferred graphene system (considering its three-layered structure) equates to about 0.4% of the total mass (<0.08 atomic %). However, given its depth localization, the copper residue appears to be spatially decoupled from the graphene overlayer and thus may not contribute to its defect density. Moreover, it is virtually undetectable by other spectroscopic techniques, for example, XPS, accounting for <0.03% of the mass (<0.006 atomic %) in the first 10 nm of the graphene/h-BN surface (details in Supplementary Note 5).

Other identified interfacial species (BS^−^ and CB^−^) are a result of chemisorbed adventitious sulfur or carbon, respectively, with h-BN and are formed before the addition of graphene. Most of the physisorbed adventitious species (for example, CH_2_^−^, Cl^−^ and OH^−^) at the graphene, h-BN and SiO_2_ surfaces are likely diffused to the sides of the graphene domains by the capillary forces exerted at the graphene/h-BN and graphene/SiO_2_ interfaces or uniformly spread at the graphene surface^15^. A special case is represented by the OH^−^ species that appears to be located in two layers, one at the very surface corresponding to the physisorbed water (due to air exposure) and another seemingly chemisorbed in the graphene overlayer (Fig. 3b). Embedded in the graphene surface and with little to no possibility of removal, the chemisorbed water arising from the transfer process is a well-known contamination issue in SPM research of graphene on various substrates^27^.

### Chemical mapping of residues

**Figure 4:**
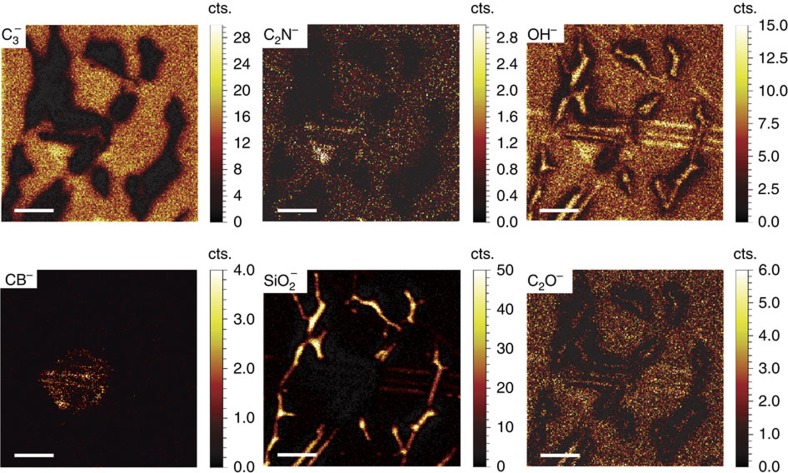
Planar distribution of the main residual species. High-resolution (burst alignment mode) TOF-SIMS maps of C_3_^−^, C_2_N^−^, OH^−^, CB^−^, SiO_2_^−^ and C_2_O^−^ secondary ions after about 0.3 nm of surface removal by Cs^+^. The C_3_^−^ signal corresponds to the graphene overlayer, C_2_N^−^ to the (NH_4_)_2_S_2_O_8_ (lift-off solvent) residues, OH^−^ to the chemisorbed water, C_2_O^−^ to the PMMA/acetone residues partial underlayer, CB^−^ to the adventitious chemisorbed carbon at the h-BN surface and SiO_2_^−^ to the substrate. The same lateral localization of the C_3_^−^, C_2_N^−^ and C_2_O^−^ secondary ion signals indicate the solvent residues are uniformly distributed, and thus chemisorbed, within the graphene overlayer and subsequent partial underlayers. The h-BN flake investigated here is different from the h-BN flakes investigated in Figs 1, 2, 3. Scale bars, 10 μm.

Figure 4 presents a series of secondary ion maps (50 × 50 μm^2^) recorded in high lateral resolution (∼200 nm) mode with a Bi_3_^+^ (30 keV) analysis ion beam. These maps are recorded after ∼0.3 nm of the surface has been removed by Cs^+^ sputtering (500 eV ion energy) and represent the main species of interest related to the graphene overlayer (C_3_^−^), chemisorbed copper solvent residues (C_2_N^−^), chemisorbed water (OH^−^), PMMA/acetone residues partial underlayer (C_2_O^−^), chemisorbed adventitious organic material at the h-BN flake surface (CB^−^) and the SiO_2_ substrate (SiO_2_^−^). The dark areas visible in the C_3_^−^ maps correspond to the space between the graphene islands that are inherent to the transfer process. Having the same in-plane localization, the C_3_^−^, C_2_N^−^, S^−^ (Supplementary Fig. 9; Supplementary Note 6) and C_2_O^−^ secondary ion signals indicate that the ammonia persulfate and PMMA/acetone residues are uniformly distributed, at ranges >200 nm (as far as the TOF-SIMS lateral resolution permits), within the transferred graphene. In addition, as the depth profiles of C_2_N^−^ and S^−^ extend over the depth profiles of C_3_^−^ and C_2_O^−^ (Supplementary Fig. 7a), we conclude that cyanide and sulfide compounds are most probably chemisorbed in the graphene layer during the copper wet-etching process and further expand into two partial adlayers, leading to a passivation effect. Finally, upon PMMA removal, the organic oxide residues most probably diffuse through the graphene overlayer or its grain boundaries and stabilize right below in the second adlayer. The CB^−^ and SiO_2_^−^ maps show the position of the h-BN flake and graphene grain boundaries (that is, exposed substrate), respectively. As a clear indication of being a PMMA/acetone marker, the C_2_O^−^map follows both the C_3_^−^ and SiO_2_^−^ maps, as expected for a solvent that was used after the deposition of the PMMA/graphene system onto the h-BN/SiO_2_ substrate. Given that the physisorbed species (∼0.3 nm) were previously removed by sputtering, the secondary ion map of the OH^−^ species represents the chemisorbed water that appears uniformly distributed within the plane of the graphene overlayer and the exposed SiO_2_ substrate. Additional contaminants, if present, were below the detection limit or their signal was too low to produce a reasonable image, as in the case of Cu^−^. Albeit impossible to spatially map in plane, the copper residue is most probably isotropically chemisorbed at the bottom of the graphene overlayer in the lower ammonia persulfate residual layer, as inferred in Fig. 3b, following the wet-etching process of the initial copper foil support.

## Discussion

To summarize, in depth AFM and TOF-SIMS analysis show that the transferred graphene system (i) is chemically inert with respect to h-BN but interacts with SiO_2_ substrate, and (ii) consists of three atomic-like layers: a single layer graphene and two partial atomic underlayers that combine residues from the solvents used in the transfer process and the initial copper foil support for the CVD graphene. All residues detected as a part of the graphene system are chemisorbed in or at the bottom of the graphene overlayer, thereby having a passivation effect and possibly acting as an electronic decoupling layer from the substrate. Located in the lower organic residual (passivation) layer, the copper residue (< 0.08 atomic %) appears spatially decoupled from the graphene overlayer thus not contributing to the actual graphene defect density. Finally, traces of water are identified both physisorbed at the very surface of the 2D system and chemisorbed in the graphene overlayer, confirming previous SPM observations^27^.

As shown above, all detected surface and interface species can be traced back to the transfer or CVD growth processes, thus TOF-SIMS profiling can directly measure the effects of synthesis, handling, and fabrication processes on the final 2D materials and heterostructures. Such detailed information could be critical towards understanding their device function. TOF-SIMS can also be applied to other ultra-thin layered structures, providing detailed, vertical chemical analysis at the atomic level (Supplementary Fig. 10 and Supplementary Note 7). We thus suggest that the methods described here will be of great importance in understanding the influence chemical composition and structure have upon multilayer-stacked ‘2D devices'. Further work is expected to enable an improved understanding of the application of TOF-SIMS to atomically-thin materials.

## Methods

### Sample preparation

The substrate was prepared by depositing mechanically exfoliated h-BN flakes from h-BN powder (acquired from Momentive Performance Materials, >99% purity) atop SiO_2_ (285 nm thick) on a Si wafer^28^. The graphene was grown on copper foil by the CVD process, as previously described^10^. For transfer, PMMA (Sigma-Aldrich) was spin-coated onto the graphene and the copper foil was dissolved in 0.5 M ammonia persulfate (((NH_4_)_2_S_2_O_8_); Sigma-Aldrich) solution^10^. The resulting PMMA-supported CVD-graphene was then placed onto the discrete h-BN on SiO_2_/Si substrate and the top PMMA was removed using acetone, giving the final graphene/h-BN/SiO_2_ configuration. We deliberately used common growth, processing and transfer techniques to produce 2D heterostructures that are typical of other published results.

### Characterization techniques

Raman spectroscopy and mapping, AFM and TOF-SIMS were used in conjunction to characterize the structure, morphology and layered chemical composition, respectively, of the graphene/h-BN/SiO_2_ system. The instrumentation consisted of a Raman spectrometer WITec alpha300 using a laser wavelength of 488 nm, an AFM Asylum Research MFP3D and a TOF-SIMS ION-TOF GmbH TOF.SIMS 5 configured with 500 eV Cs^+^ sputtering and 30-keV Bi_1_^+^/Bi_3_^+^ analysis ion beams. Depending on the acquisition mode, the TOF-SIMS analysis ion gun was set in either high-current bunched mode (Bi_1_^+^ at 30 keV ion energy and 18 ns pulse duration) for depth profiling or burst alignment mode (Bi_1_^+^/Bi_3_^+^ at 30 keV ion energy and pulse duration of 100 ns without or 18 ns with bursts) for high lateral resolution. The raster area, beam sample currents and specific settings for both the analysis and sputtering beams are indicated in the main text and supporting information. The high-current and burst alignment (with bursts) modes allowed a mass resolution better than 5,000 (*m*/δ*m*) for all analysed masses. All detected secondary ions had negative polarity. The TOF-SIMS data were acquired at a base pressure of ∼7.5 × 10^−10^ Torr.

## 

## Additional information

**How to cite this article:** Chou, H. *et al.* Revealing the planar chemistry of two-dimensional heterostructures at the atomic level. *Nat. Commun.* 6:7482 doi: 10.1038/ncomms8482 (2015).

## Supplementary Material

Supplementary Information Supplementary Figures 1-10, Supplementary Notes 1-7 and Supplementary References

## References

[b1] Novoselov K. S. *et al.* Electric field effect in atomically thin carbon films. Science 306, 666–669 (2004). 15499015 10.1126/science.1102896

[b2] Butler S. *et al.* Progress, challenges, and opportunities in two-dimensional materials beyond graphene. ACS Nano 7, 2898–2926 (2013). 23464873 10.1021/nn400280c

[b3] Chhowalla M. *et al.* The chemistry of two-dimensional layered transition metal dichalcogenide nanosheets. Nat. Chem. 5, 263–275 (2013). 23511414 10.1038/nchem.1589

[b4] Geim A. K. & Grigorieva I. V. Van der Waals heterostructures. Nature 499, 419–425 (2013). 23887427 10.1038/nature12385

[b5] Hsu A. *et al.* Large-area 2-D electronics: materials, technology, and devices. Proc. IEEE 101, 1638–1652 (2013).

[b6] Lemme M. C., Li L.-J., Palacios T. & Schwierz F. Two-dimensional materials for electronic applications. MRS Bull. 39, 711–718 (2014).

[b7] Eisenstein J. P. & Macdonald A. H. Bose-Einstein condensation of excitons in bilayer electron systems. Nature 432, 691–694 (2004). 15592403 10.1038/nature03081

[b8] Bonaccorso F. *et al.* Production and processing of graphene and 2d crystals. Mater. Today 15, 564–589 (2012).

[b9] Geim A. K. & Novoselov K. S. The rise of graphene. Nat. Mater. 6, 183–191 (2007). 17330084 10.1038/nmat1849

[b10] Li X. *et al.* Large-area synthesis of high-quality and uniform graphene films on copper foils. Science 324, 1312–1314 (2009). 19423775 10.1126/science.1171245

[b11] Wang L., Chen Z., Dean C. R. & Taniguchi T. Negligible environmental sensitivity of graphene in a hexagonal Boron Nitride/Graphene/h-BN Sandwich structure. ACS Nano 6, 9314–9319 (2012). 23009029 10.1021/nn304004s

[b12] Pirkle A. *et al.* The effect of chemical residues on the physical and electrical properties of chemical vapor deposited graphene transferred to SiO2. Appl. Phys. Lett. 99, 122108 (2011).

[b13] Chen J.-H., Jang C., Xiao S., Ishigami M. & Fuhrer M. S. Intrinsic and extrinsic performance limits of graphene devices on SiO2. Nat. Nanotechnol. 3, 206–209 (2008). 18654504 10.1038/nnano.2008.58

[b14] Garcia A. G. F. *et al.* Effective cleaning of hexagonal boron nitride for graphene devices. Nano Lett. 12, 4449–4454 (2012). 22866696 10.1021/nl3011726

[b15] Haigh S. J. *et al.* Cross-sectional imaging of individual layers and buried interfaces of graphene-based heterostructures and superlattices. Nat. Mater. 11, 764–767 (2012). 22842512 10.1038/nmat3386

[b16] Elko-Hansen T. D. M., Dolocan A. & Ekerdt J. G. Atomic interdiffusion and diffusive stabilization of cobalt by copper during atomic layer deposition from bis (N-tert-butyl-N'-ethylpropionamidinato) cobalt (II). J. Phys. Chem. Lett. 5, 1091–1095 (2014). 26274454 10.1021/jz500281k

[b17] Li Q. *et al.* Growth of adlayer graphene on Cu studied by carbon isotope labeling. Nano Lett. 13, 486–490 (2013). 23278710 10.1021/nl303879k

[b18] Dean C. R. *et al.* Boron nitride substrates for high-quality graphene electronics. Nat. Nanotechnol. 5, 722–726 (2010). 20729834 10.1038/nnano.2010.172

[b19] Ismach A. *et al.* Toward the controlled synthesis of hexagonal boron nitride films. ACS Nano 6, 6378–6385 (2012). 22702240 10.1021/nn301940k

[b20] Li X. *et al.* Graphene films with large domain size by a two-step chemical vapor deposition process. Nano Lett. 10, 4328–4334 (2010). 20957985 10.1021/nl101629g

[b21] Ferrari A. C. *et al.* Raman spectrum of graphene and graphene layers. Phys. Rev. Lett. 97, 187401 (2006). 17155573 10.1103/PhysRevLett.97.187401

[b22] Ferrari A. C. & Basko D. M. Raman spectroscopy as a versatile tool for studying the properties of graphene. Nat. Nanotechnol. 8, 235–246 (2013). 23552117 10.1038/nnano.2013.46

[b23] Li X., Cai W., Jung I. & An J. Synthesis, characterization, and properties of large-area graphene films. ECS Trans. 19, 41–52 (2009).

[b24] Iltgen K. Optimized time-of-flight secondary ion mass spectroscopy depth profiling with a dual beam technique. J. Vac. Sci. Technol. A 15, 460 (1997).

[b25] Zimmerman J. D. *et al.* Control of interface order by inverse quasi-epitaxial growth of squaraine/fullerene thin film photovoltaics. ACS Nano 7, 9268–9275 (2013). 23991668 10.1021/nn403897d

[b26] Hofmann S. Profile reconstruction in sputter depth profiling. Thin Solid Films 398–399, 336–342 (2001).

[b27] Xue J. *et al.* Scanning tunnelling microscopy and spectroscopy of ultra-flat graphene on hexagonal boron nitride. Nat. Mater. 10, 282–285 (2011). 21317900 10.1038/nmat2968

[b28] Lee G.-H. *et al.* Electron tunneling through atomically flat and ultrathin hexagonal boron nitride. Appl. Phys. Lett. 99, 243114 (2011).

