# Corrigendum: Revealing the planar chemistry of two-dimensional heterostructures at the atomic level

**DOI:** 10.1038/ncomms9229

**Published:** 2015-08-21

**Authors:** Harry Chou, Ariel Ismach, Rudresh Ghosh, Rodney S. Ruoff, Andrei Dolocan

*Nature Communications* 6, Article number: 7482 (2015); 10.1038/ncomms8482 Published 06232015; Updated 08212015

The financial support for this Article was not fully acknowledged. The Acknowledgements should have included the following: The atomic force microscope used in these studies was acquired under the program ‘Understanding Charge Separation and Transfer at Interfaces in Energy Materials (EFRC:CST)', an Energy Frontier Research Center funded by the U.S. Department of Energy, Office of Science, Office of Basic Energy Sciences under Award Number DE-SC0001091.

