# Supplementary material for: Revealing the planar chemistry of two-dimensional heterostructures at the atomic level
Source: Nat Commun. 2015 Jun 23;6:7482. doi: 10.1038/ncomms8482 (PMC4557291; doi:10.1038/ncomms8482)
Supplement: Supplementary Information — Supplementary Figures 1-10, Supplementary Notes 1-7 and Supplementary References [file ncomms8482-s1.pdf]

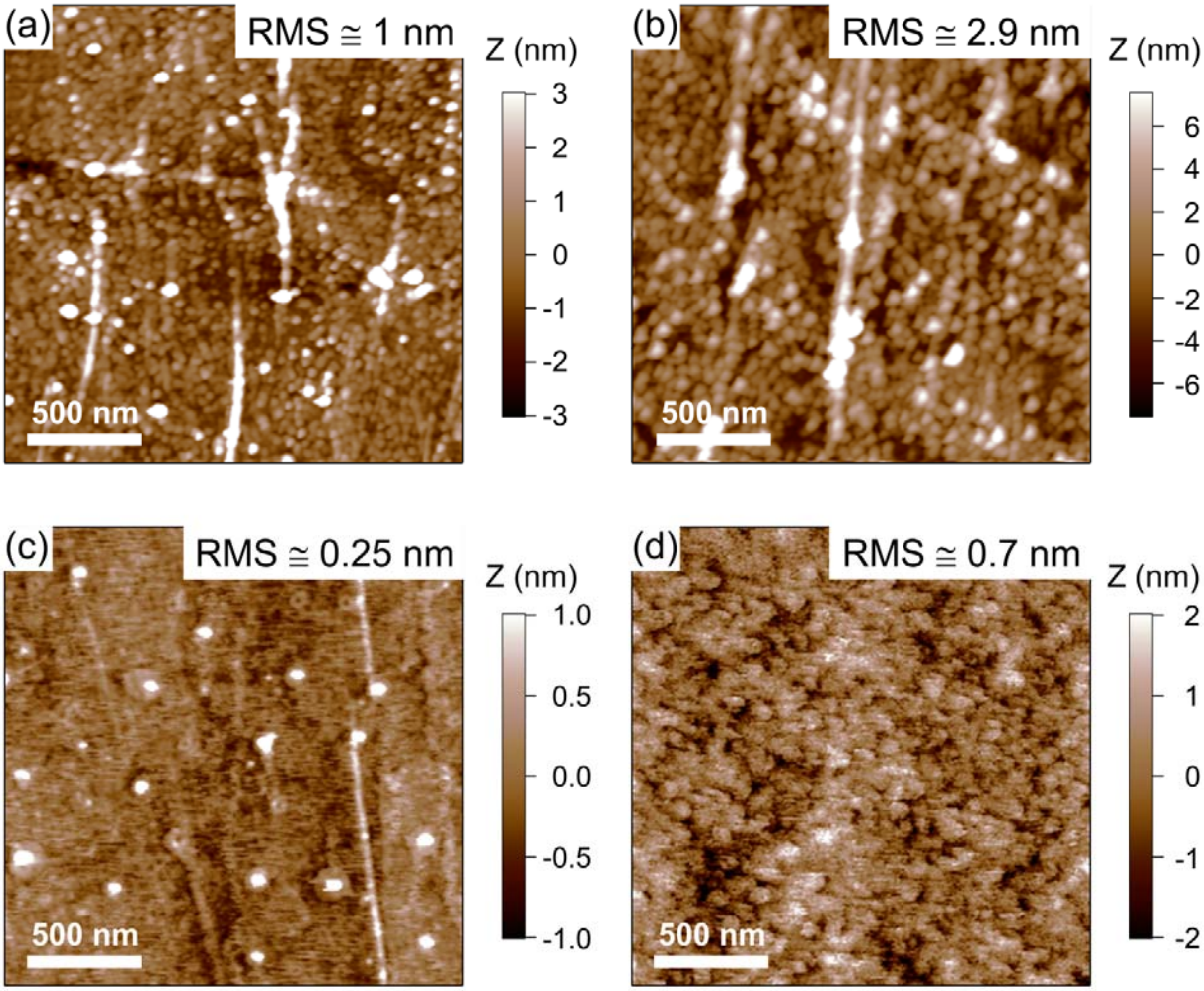

Supplementary Figure 1: AFM topography of the graphene/SiO_2 [(a) and (c)] and graphene/h-BN [(b) and (d)] surfaces acquired before [(a) and (b)], and after [(c) and (d)], respectively, 35 seconds of Cs^+ (500 eV ion energy, 45 nA sample current, sputtering area of 250 x 250 μm^2) sputtering. The surface corrugation RMS is reduced by a factor of ~4, from ~1 nm to ~0.25 nm and from ~2.9 nm to ~ 0.7 nm in the case of graphene covered SiO_2 and h-BN, respectively, following the Cs^+ sputtering.

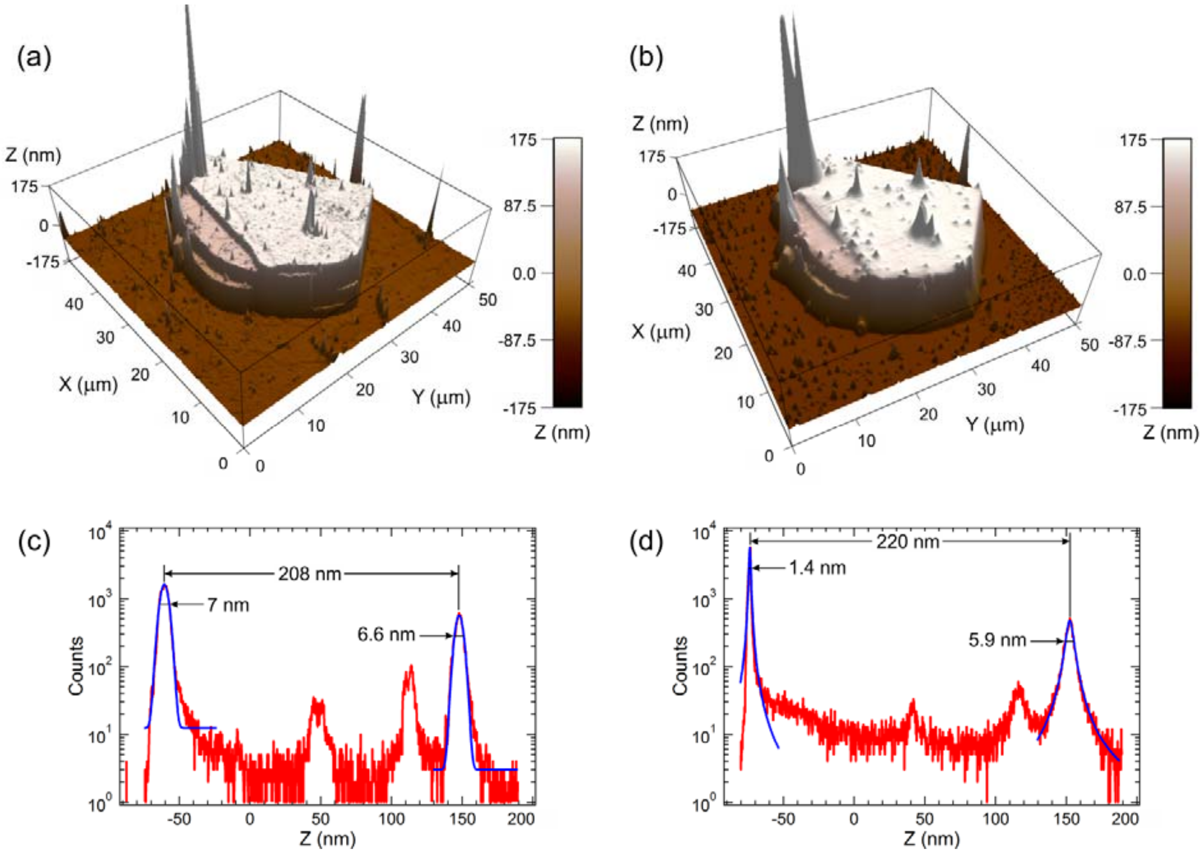

Supplementary Figure 2. (a) and (b) AFM height topography of an h-BN flake covered by graphene obtained before and after 35 s of Cs^{+} (500 eV ion energy, 45 nA sample current, sputtering area of 250 x 250 μm^{2}) sputtering. (c) and (d) Height distributions (i.e., histograms) of the AFM maps of (a) and of (b), respectively. Sputtering induces a strong reduction of surface corrugation as inferred by the change in shape and width of the two main histogram features.

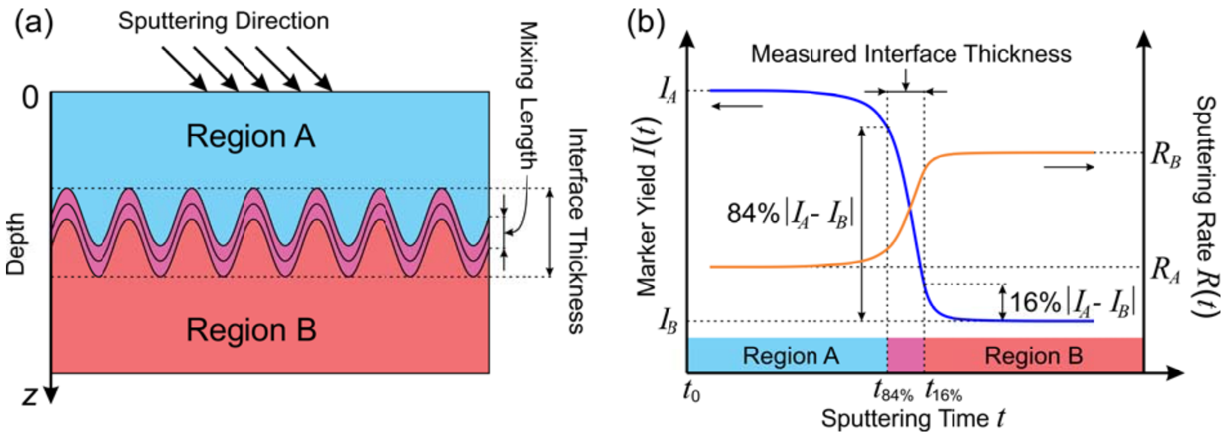

Supplementary Figure 3. (a) Schematic of an interface between two materials, A and B, that consists of atomic mixing and corrugation, the latter represented here by the RMS of the roughness. (b) Depth profiling through such interface adds sputtering effects that, together with corrugation, can be deconvoluted from the measured interface thickness to give the atomic mixing length.

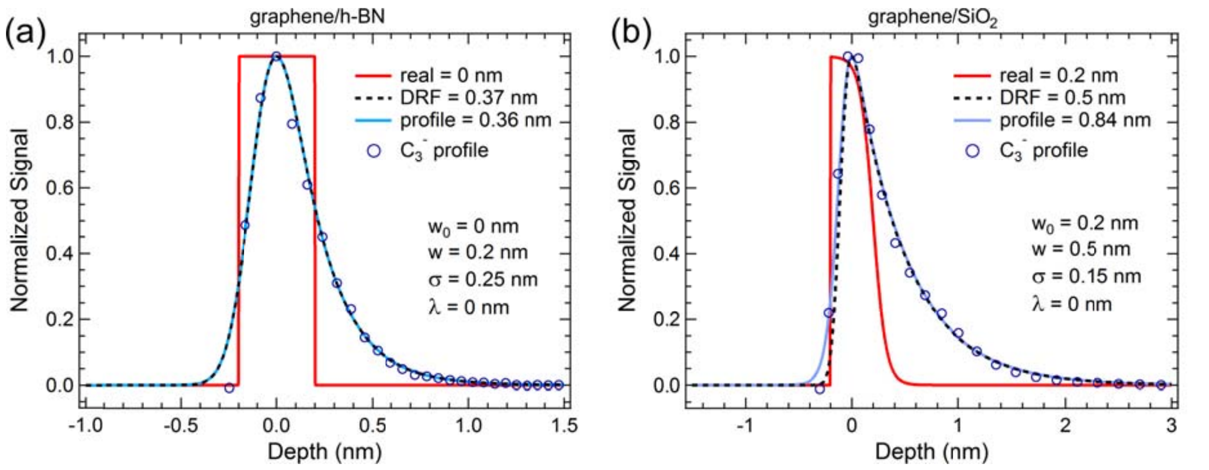

Supplementary Figure 4. (a) Simulation of the measured graphene/h-BN (a) and graphene/SiO_2 (b) C_3^- interface profiles. All curves are centered at z = 0 for clarity.

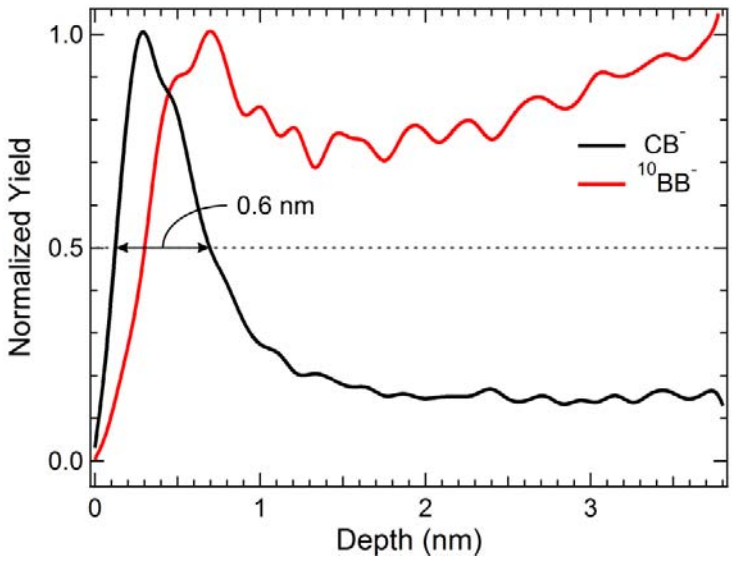

Supplementary Figure 5: Surface depth profile of a h-BN flake on top of SiO_2/Si substrate. The $^{10}BB^-$ species represents the h-BN flake while CB^- the chemisorbed species at the h-BN surface. Sputtering conditions: analysis ion beam: Bi_1^+ (HC mode, 30 keV ion energy, ~3 pA sample current, probing area of 100 x 100 µm^2); sputtering ion beam: Cs^+ (500 eV ion energy, ~45 nA sample current, sputtering area of 200 x 200 µm^2).

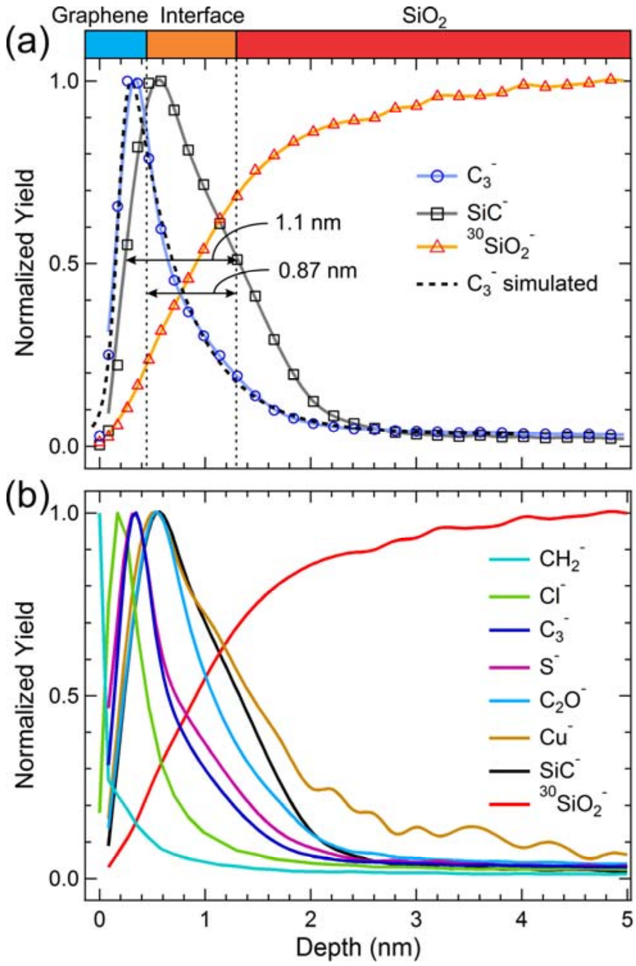

Supplementary Figure 6: Depth profile of graphene on SiO_2/Si substrate. (a) The graphene profile, represented by the C_3^- species, the substrate represented by $^{30}SiO_2^-$ species, and the interfacial region represented by the SiC^- species. The simulated C_3^- profile (from Supplementary Fig. 4b) is also appended. The discrete markers represent the actual TOF-SIMS depth profiling data whereas the continuous lines the 1-point spline interpolations. (b) Additional profiles which are attributed to the graphene synthesis (Cu^-) and transfer processes (S^- and C_2O^-). The graphene profile shows some chemical interaction with the substrate. Sputtering conditions: analysis ion beam: Bi_1^+ (HC mode, 30 keV ion energy, ~3 pA sample current, probing area of 100 x 100 μm^2); sputtering ion beam: Cs^+ (500 eV ion energy, ~45 nA sample current, sputtering area of 200 x 200 μm^2).

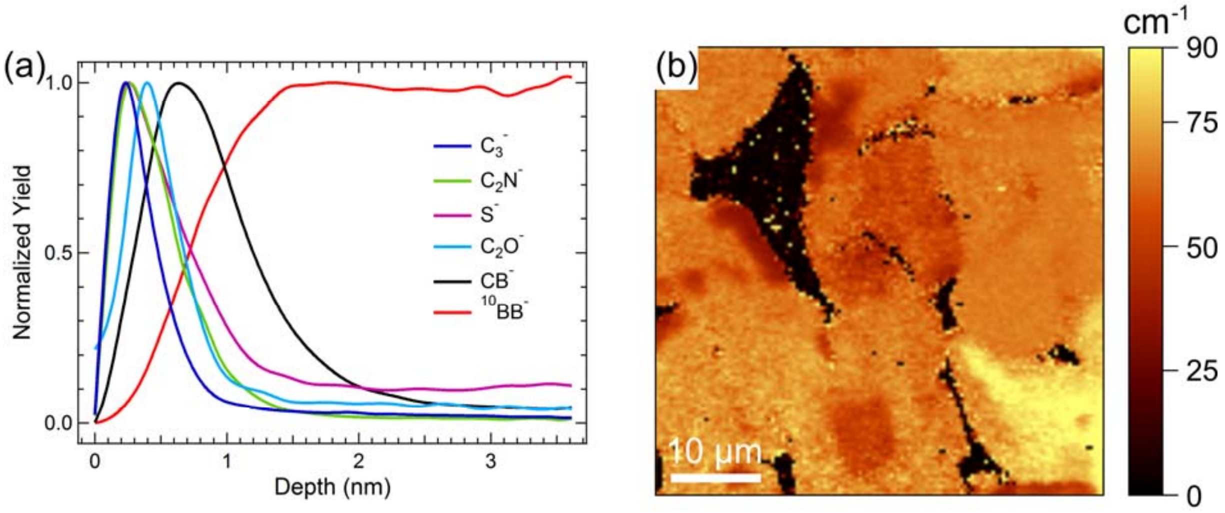

Supplementary Figure 7. (a) Graphene/h-BN interface. S^- and C_2N^- species show similar depth localization (peak position) with C_3^-, indicating that the lift-off solvent, $(NH_4)_2S_2O_8$, is chemically interacting with the graphene layer. Sputtering conditions: analysis ion beam: Bi_1^+ (HC mode, 30 keV ion energy, ~3 pA sample current, probing area of 100 x 100 µm^2); sputtering ion beam: Cs^+ (500 eV ion energy, ~45 nA sample current, sputtering area of 200 x 200 µm^2). (b) FWHM map of the characteristic Raman G peak of graphene. The Raman shift spectral map is recorded at the same location as in Fig. 1 in the main text. The FWHM variation of the G peak indicates doping.

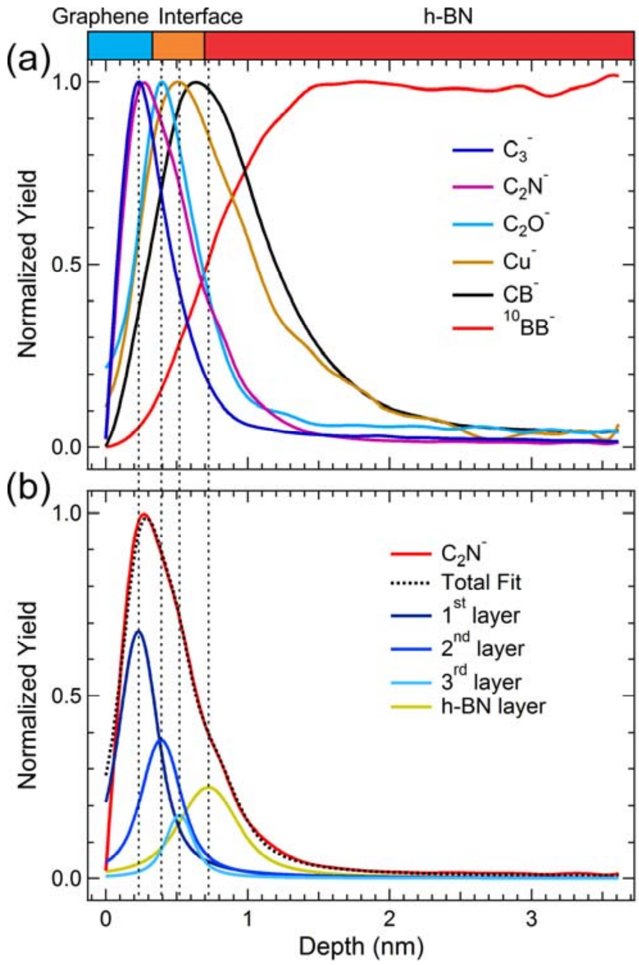

Supplementary Figure 8. (a) The 3 layer structure of the transferred graphene system represented by the C_3^- (graphene overlayer), C_2N^-, C_2O^- and Cu^- (residual layers from the transfer process) markers. (b) Fit of the C_2N^- profile with 4 Voigt functions, the first 3 representing the transferred graphene system while the fourth the adventitious organic material that is already chemisorbed at the h-BN surface before the transfer process. Sputtering conditions: analysis ion beam: Bi_1^+ (HC mode, 30 keV ion energy, ~3 pA sample current, probing area of 100 x 100 µm^2); sputtering ion beam: Cs^+ (500 eV ion energy, ~45 nA sample current, sputtering area of 200 x 200 µm^2).

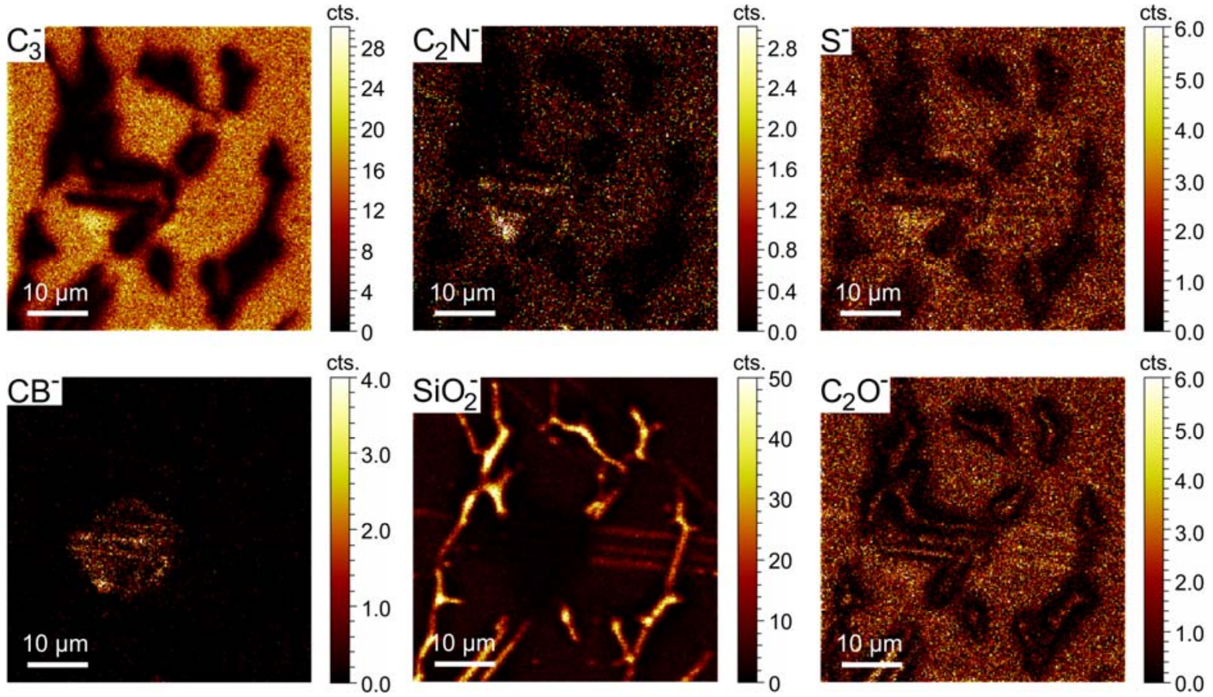

Supplementary Figure 9. High resolution (BA mode) TOF SIMS maps of C_3^-, C_2N^-, S^-, CB^-, SiO_2^-, and C_2O^- secondary ions after about 0.3 nm of surface removal by Cs^+. C_3^- signal corresponds to the graphene overlayer, C_2N^- and S^- to the lift-off solvent, $(NH_4)_2S_2O_8$, residues, C_2O^- to the PMMA/acetone residues partial underlayer, CB^- to the adventitious chemisorbed carbon at the h-BN surface and SiO_2^- to the substrate. By showing the same lateral localization; the C_3^-, C_2N^-, C_2O^-, and S^- secondary ion signals indicate that the solvent residues are uniformly distributed, and thus chemisorbed, within the graphene overlayer and subsequent partial underlayers. Sputtering conditions: analysis beam: Bi_3^+ (BA mode with 7 bursts, 30 keV ion energy, ~40 fA sample current, probing area of 50 x 50 µm^2); sputtering beam: Cs^+ (500 eV ion energy, ~45 nA sample current, sputtering area of 250 x 250 µm^2).

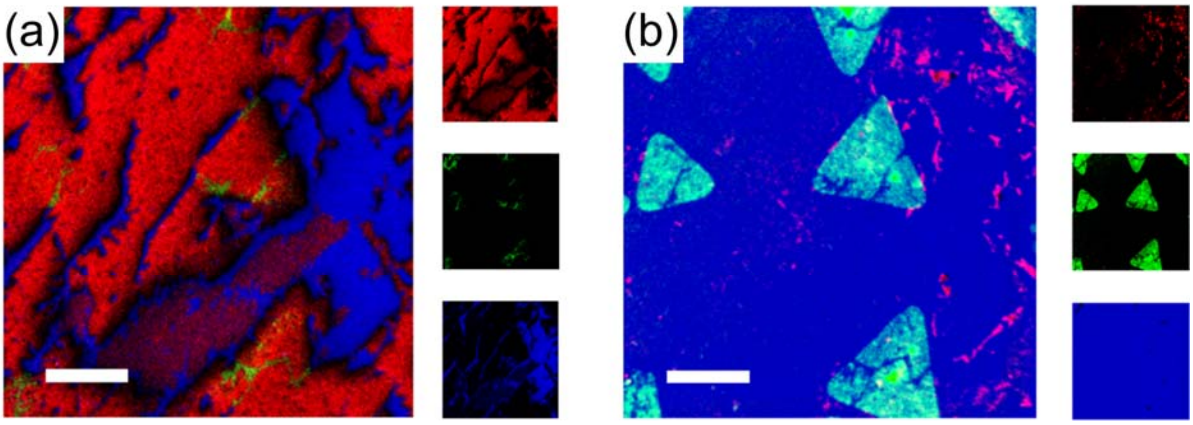

Supplementary Figure 10. TOF SIMS high resolution (BA) maps of the graphene/MoS_2 heterostructure in its initial state (a) and after 50 s of sputtering (b). The instrument conditions were a 30 keV Bi^+ analysis ion beam and a 1 keV Cs^+ sputtering ion beam. The graphene layer is controllably sputtered away to reveal the underlying MoS_2 islands. The scale bars are 20 µm.

Supplementary Note 1. AFM height topography of an h-BN flake and SiO_2 substrate covered by graphene islands before and after Cs^+ sputtering

The secondary ions collected during depth profiling are subject to the morphology and the electronic properties of the surface from which they are sputtered. In particular, the initial roughness and any roughness induced through sputtering (Supplementary Fig. 1) will affect the depth resolution of collected profiles. In order to (i) understand the physical effects of sputtering on the graphene/h-BN and graphene/SiO2 interfaces, and (ii) estimate sputtering rates for the species of interest, the surface topography of the 2D heterostructure was investigated by AFM before (Supplementary Fig. 2a) and after (Supplementary Fig. 2b) 35 seconds of sputtering with Cs^+ (500 eV ion energy, 45 nA sample current, sputtering area of 250 x 250 μm^2). Both height distributions (histograms) shown in the Supplementary Fig. 2c and 2d, corresponding to the height topographies in Supplementary Fig. 2a and 2b, respectively, exhibit two main peaks attributed to the SiO_2 substrate ($Z \approx -65$ nm) and h-BN flake surface ($Z \approx 150$ nm). An obvious change in shape from Gaussian to Lorentzian and a strong reduction of the width (full width at half maximum, FWHM) for the two main histogram peaks suggest that Cs^+ sputtering substantially decreases the surface corrugation of both SiO_2 substrate and h-BN flake surface. As inferred from the maps in Supplementary Fig. 1, sputtering reduces the overall corrugation (defined as the root mean square (RMS) or standard deviation of the height distribution and proportional to its FWHM) by a factor of ~4, from ~2.9 nm to ~0.7 nm for the h-BN flake and from ~1 nm to ~0.25 nm for the SiO_2. Albeit visible in the histograms (Supplementary Fig. 2c and 2d), the sputtering-induced FWHM reduction of the second peak by only 10 % (from ~6.6 nm to ~5.9 nm, based on Gaussian and Lorentzian fits indicated in blue, respectively) can be accounted for by the lack of long range flatness of the h-BN flake top surface which supports several terraces and large out of plane features (Supplementary Fig. 2a and 2b). In contrast, the first peak of the histograms shows a factor of ~5 decrease in FWHM (from ~7 nm to ~1.4 nm) following the sputtering process, comparable with the result extracted from the local AFM maps (Supplementary Fig. 1), due to the intrinsic long range flatness of the Si wafer. During depth profiling, the corrugation at the regressing surface, as the interface between graphene and h-BN is exposed, is considered to be an average of the corrugation measured before and after sputtering[1]. Complete removal of the h-BN flake (~215 nm) and SiO_2 (~285 nm) films reveals sputtering rates of about 0.04 nm s^{-1} and 0.14 nm s^{-1}, respectively, when sputtering areas of 250 x 250 μm^2 with Cs^+ at 500 eV ion energy. Assuming single layer graphene atop the h-BN flake, as proven by Raman spectroscopy and mapping (Fig. 1a, b, and c in the main text), the sputtering rate of graphene reads ~0.06 nm s^{-1}. The h-BN flake height difference (i.e., spacing between the two major histogram peaks in Supplementary Fig. 2c and 2d, ~208 and ~220 nm, respectively) reported by AFM following 35 seconds of Cs^+ sputtering

can be attributed to large disparities in h-BN and SiO_2 sputtering rates and long range surface corrugation. For depth profiling, a Cs^+ (500 eV ion energy) sputtering area of 200 x 200 μm^2 was used. The sputtering rates of graphene and h-BN were estimated, in this case, at 0.09 nm s^{-1} and 0.06 nm s^{-1}, respectively, given the rates calculated previously and linearity between removal rate of material and Cs^+ beam areal dose density, which is inversely proportional with the sputtered area[2].

Supplementary Note 2. TOF-SIMS sputtering time to depth conversion

Time of flight secondary ion mass spectrometry (TOF-SIMS) is a highly chemically sensitive and surface sensitive analytical technique which bombards the sample with a low dose of analysis ion pulses (i.e. analysis ion beam) and analyzes the resulting partially (<1 %) ionized debris (i.e., secondary ions, SI) through a time of flight technique. Besides simple spectroscopy, TOF-SIMS can provide high resolution imaging (~70 nm at best in our case), while detecting the masses of interest in parallel, and depth profiling with <1 nm depth resolution by adding a sputtering ion beam that has a much higher intensity than the analysis ion beam and which is used to remove material from the sample while the analysis ion beam probes the regressing surface. The analysis ion beam consists of high energy (30 keV) Bi_1^+ or Bi_3^+ ion pulses (18 ns) with a measured sample current of ~3 pA or 0.9 pA, respectively, typically raster scanned over a 100 x 100 μm^2 probing area. For depth profiling, the analysis ion beam probing area was centered within a 200 x 200 μm^2 or 250 x 250 μm^2 regressing area that was sequentially sputtered by a sputtering ion beam (Cs^+ at 500 eV and ~45 nA measured sample current) during the data acquisition. The average instrument operating pressure was 7.5 x 10^{-10} Torr and the analyzer was biased to collect negative SI. Depth profiling was performed on both graphene/h-BN/SiO_2 and graphene/SiO_2 structures.

The sputtering rates for graphene and h-BN were calculated based on the thicknesses (determined by AFM and Raman) and the corresponding times needed to sputter through the respective film layers. For converting the sputtering time, t, into a depth, z, a rate model assuming the instantaneous sputtering rate, $R(t)$, at the interface of two films (referred to herein as A and B) as a linear combination of the individual sputtering rates was used[1]:

$$R(t) = \left|\frac{I(t) - I_B}{I_A - I_B}\right| R_A + \left|\frac{I(t) - I_A}{I_A - I_B}\right| R_B = \dot{z}$$

where $I(t)$ is the normalized secondary ion yield of a species representing the material A or B, I_A and I_B are the values of $I(t)$ in the materials A and B, respectively, and $R_A$and R_B are the

individual sputtering rates for A and B, respectively (Supplementary Fig. 3). The linear coefficients are essentially proportional to the molar fractions of the two materials[2] at the sputtering time, t. Thus, the sputtering depth, $z(t)$ corresponding to sputtering time, t, is expressed as:

$$z(t) = \int_{t_0}^{t} dt' R(t')$$

where t_0 is the initial sputtering time. Application of this model on the C_3^- marker for the graphene/h-BN interface allows the conversion $t \rightarrow z(t)$. The polyatomic species C_3^- and $^{10}BB^-$ were selected as markers for the bulk graphene and h-BN, respectively, to avoid intrinsic carbon and BN artifact signals due to residues, oxides or surface-adsorbed species.

Supplementary Note 3. The mixing-roughness-information (MRI) model

The mixing-roughness-information (MRI) model was employed to determine the atomic mixing at the graphene/h-BN and graphene/SiO_2 interfaces following TOF-SIMS depth profiling[2,3]. For a given interface between two materials this model is based on three major assumptions: (1) the real interface (equivalent with the intrinsic atomic mixing between the two materials following the fabrication) will appear broadened upon depth profiling due to three phenomenological factors: (a) sputtering induced atomic mixing, (b) intrinsic and sputtering induced corrugation and (c) actual signal depth of origin (equivalent with the escape depth of the analyzed particle) at the regressing surface; (2) these three factors can be disentangled and considered as independent from each other, and (3) they can be described by analytical functions of depth whose convolution defines the so called depth resolution function (DRF). By deconvoluting the DRF from the measured interface thickness obtained by depth profiling one can extract the real interface thickness (i.e. the fabrication induced (or real) atomic mixing length, Supplementary Fig. 3a). Following the work of Hofmann[3] the functions representing the mixing, roughness and information factors can be written as:

$$g_w(z - z_0) = A_w \exp\left[-\frac{(z - z_0 + w)}{w}\right] \theta(z - z_0 + w)$$

$$g_\sigma(z - z_0) = A_\sigma \exp\left[-4\ln(2)\frac{(z - z_0)^2}{\sigma^2}\right]$$

$$g_\lambda(z - z_0) = A_\lambda \exp\left[\frac{(z - z_0)}{\lambda}\right] \theta(z_0 - z)$$

where z is the sputtered depth, z_0 is the running depth for which the contributions are calculated, A_w, A_σ and A_λ are some normalization constants such that $\int_{-\infty}^{\infty} dz\, g_{w,\sigma,\lambda}(z) = 1$, w, σ and λ are the mixing, roughness and information parameters, respectively, and $\theta(z)$ is the Heaviside step function (equal with 1 if $z \geq 0$ and 0 otherwise). The mixing and information parameters, w and λ, represent the length to which their respective contributions (g_w and g_λ, respectively) drop by a factor of $1/e$. The full width at half maximum of the corrugation contribution, g_σ, that is σ, represents the RMS of the corrugation at the z_0 plane. The DRF reads then:

$$g_{DRF}(z) = \int_{-\infty}^{\infty} dz' \int_{-\infty}^{\infty} dz''\, g_w(z' - z'') g_\sigma(z'') g_\lambda(z - z')$$

Finally, the normalized depth profile of a certain species (to the maximum secondary ion intensity, I_0) can be written as:

$$\frac{I(z)}{I_0} = \int_{-\infty}^{\infty} dz' g_{DRF}(z' - z) \chi(z')$$

where $\chi(z)$ represents the molar fraction of the species at the depth z, i.e. the species' *real* normalized profile. For a given interface represented by the edge of the profile $I(z)$ (see Supplementary Fig. 3) the depth comprised between the 84.13 % and 15.87 % of the $I(z)$ edge height provides the measured interface thickness. Consequently, after the DRF deconvolution from $I(z)$, the depth comprised between the 84.13 % and 15.87 % of the resulting $\chi(z)$ edge provides the fabrication induced atomic mixing length (i.e. the *real* atomic mixing length). The 84 to 16 % levels are standard in the SIMS community but are meaningful only if the DRF is a Gaussian.

To simulate the measured (normalized to the maximum) graphene profiles (Fig. 3 and Supplementary Fig. 6) we use the forward calculation procedure[3] where we start by assuming the real C_3^- profile of the form $\chi(z) = \theta(z - z_0 + d_{gr})\{1 + \exp[3.33587(z - z_0)/w_0]\}^{-1}$, with d_{gr} the graphene thickness (assumed to be a monolayer, i.e. 0.4 nm) and w_0 the real atomic mixing length defined above, and further convolute it with a DRF obtained by convoluting the mixing, roughness and information contributions for various w, σ and λ parameters, respectively (Supplementary Fig. 4). The resulting profile (normalized to maximum) is then fitted to the actual data points of the measured C_3^- profile (normalized to maximum) of either the graphene/h-BN or graphene/SiO_2 system. The fitting procedure searches for the minimum of the total absolute deviation in the vertical direction from the measured profile points while varying all parameters in steps of ± 0.01 nm from their starting values. In each of the two cases

the C_3^- profile was reasonably well simulated, as shown in Supplementary Fig. 4, for two different sets of w_0, w, σ and λ parameters. The function describing the real profile of the C_3^- species represents a perfect, box-like monolayer graphene, modulated by a sigmoid interface with the substrate on the right-hand side. We will discuss the results of these simulations in Supplementary Note 4.

At this stage, we emphasize a note of caution: atomic mixing and roughness cannot be completely disentangled. Within the MRI model, however, these two quantities are considered to be independent, thus one must clearly define the roughness such that it is completely separated from atomic mixing. In this case, we define the roughness as the RMS roughness given by a scanning probe microscopy tool, i.e. AFM. More often the roughness is measured before and after a depth profile through an interface.

Supplementary Note 4. Depth profiles of pristine h-BN, graphene/SiO_2, and graphene/h-BN

One way to extract the measured interface thickness from the depth profile of an ideal heterostructure[1,4,5] that consists of purely two materials is to calculate the full width at half maximum (FWHM) of a combined species (in our case CB^- or SiC^-) depth profile. In reality, however, the manufacturing process of a heterostructure adds contaminants at the interface(s) which renders this procedure inaccurate. As a result, one can represent an interface between two materials by either edge of their measured depth profiles[6]. Obviously, this process might not lead to the same result for the measured interface thickness, depending heavily on the amount of contamination and its degree of interaction with either one of the two materials. As our main interest is to understand the atomic mixing of the graphene overlayer we focus on the right-hand side edge of the C_3^- profile which we consider to be representative for the graphene interface with either the h-BN or SiO_2 substrates. This also keeps the calculation method of the measured interface thickness consistent throughout the text. By applying the forward calculation in the MRI model presented in Supplementary Note 3, a set of real atomic mixing length (w_0 < 0.01 nm), mixing (w = 0.2 nm), roughness (σ = 0.25 nm) and information (λ < 0.01 nm) parameters was obtained such that the total absolute deviation with respect to the C_3^- profile points was at minimum (Supplementary Fig. 4a). For these parameters the FWHM of the DRF reads 0.37 nm while the measured interface thickness 0.36 nm, very close to the value obtained by taking the depth in between the 84 and 16 % levels of the edge of the C_3^- interpolated curve (i.e. 0.38 nm, see Fig. 3a in the main text). Therefore, we conclude that the real atomic mixing between graphene and h-BN is negligible (w_0 < 0.01 nm), thus the graphene

overlayer is chemically inert with respect to the h-BN substrate. Additional evidence of non-mixing is provided by the CB^- profile of pristine h-BN (Supplementary Fig. S5) which yields a similar mass (i.e., total SI count) and a slightly smaller measured FWHM (~0.6 nm) than in the case of the graphene/h-BN system, implying the adventitious carbon is chemisorbed at the h-BN surface before the graphene transfer process. As TOF-SIMS is a destructive technique, combined species, other than with the sputtering species, can be created with very low yield probability (far under the TOF-SIMS detection limit), thus any detected mixed boron (or silicon) species (other than with Cs; Bi being too low in concentration to produce any detectable binding) should originate from a chemical bond (i.e. the CB^- or SiC^- species represent the chemisorbed organic material at the h-BN or SiO_2 surface, respectively).

By comparison, the graphene/SiO_2 system presents a measured interface length of ~0.87 nm (84 to 16 % of the C_3^- interpolated normalized profile, Supplementary Fig. 6a) which is comparable with the FWHM of the SiC^- profile, ~1.1 nm. In this case, the application of the forward calculation for the C_3^- profile in the MRI model yields a nonzero real atomic mixing length (w_0 = 0.2 nm), mixing (w = 0.5 nm), roughness (σ = 0.15 nm) and information (λ < 0.01 nm) parameters, thereby suggesting that the transferred graphene interacts at atomic level with the supporting SiO_2 surface (i.e., about half of the graphene thickness is atomically mixed). The simulated C_3^- profile interface thickness (0.84 nm) is close to the measured interface thickness (~0.87 nm). Compared to the graphene/h-BN case, the sputtering-induced mixing length, w, is 2.5 times larger, a consequence of the ~2.5 times higher sputtering rate of the SiO_2 substrate with respect to the graphene overlayer which allows for a deeper sputtering induced mixing of the graphene with the softer substrate. This result is based on the assumption the graphene monolayer structure is preserved within the few tens of microns patches (from which the depth profiles are extracted) comprised in the 100 x 100 μm^2 TOF-SIMS probing area; as indicated by the Raman data shown in Fig. 1b in the main text. Further evidence of mixing between graphene and SiO_2 is given by the same depth localization of the chemisorbed species in the transferred graphene system (represented by S^-, C_2O^- and Cu^-) and the chemisorbed organic species at the SiO_2 surface (represented by SiC^-), as shown in Supplementary Fig. 6 (a discussion of the physisorbed vs. chemisorbed species is presented in Supplementary Note 5). We think that, essentially, the chemisorbed species (identified below) accumulated in graphene during the transfer processes are chemically interacting with the SiO_2 substrate thus producing a mixing effect. Moreover, the measured interface thickness of the transferred graphene with the SiO_2 substrate (~0.87 nm) matches closely the measured thickness of the chemisorbed residues (see Supplementary Note 5 and Supplementary Fig. 6b).

The simulated roughness at the graphene/h-BN interface (σ = 0.25 nm) is significantly smaller than its AFM-measured roughness after sputtering (~7nm, Supplementary Fig. 1d) most probably due to large variations between the surface corrugation of different h-BN flakes; the

AFM measurements were performed on other h-BN flake than the one used to record the graphene/h-BN depth profiles to minimize the possible AFM-induced defects and contamination at the graphene surface. On the other hand, the simulated roughness of the graphene/SiO_2 interface (σ = 0.15 nm) is reasonably close to its measured roughness by AFM after sputtering (~0.25 nm, Supplementary Fig. 1c), a consequence of the long range uniform morphology of the Si/SiO_2 wafer.

Supplementary Note 5. Quantification of transfer residues. Physisorbed vs. chemisorbed species

Residuals from the transfer process at the graphene/h-BN interface include S^-, C_2N^-, C_2O^- and Cu^- species. Albeit in small amounts, S^- and C_2N^- species show the same depth profile localization as C_3^- thus indicating a chemical interaction between graphene and the lift-off solvent, $(NH_4)_2S_2O_8$ (Supplementary Fig. 7a). We consider these species (S^-, C_2N^-, C_2O^- and Cu^-) to originate from chemisorbed species since they appear right under the graphene overlayer (represented by the C_3^- marker) in the depth profile. If they were originating from physisorbed species they would easily migrate towards the edges of the graphene islands, therefore should appear before or at the same depth with the graphene overlayer. Moreover, they have similar in-plane localization as the graphene islands (except the Cu^- signal which is too weak to produce a map, see Supplementary Note 6) rather than a higher concentration along the graphene edges. In addition, besides a small D peak which is an indicator for crystal disorder, crystal grain edges, and/or heteroatom bonding to graphene (Fig. 1c in the main text), the FWHM, as well as the peak position (not shown), in-plane spatial variation (Supplementary Fig. 7b) of the characteristic Raman peak G of graphene indicates doping (i.e. chemically interacting contamination)[7]. In contrast, adventitious species like CH_2^-, Cl^-, OH^-, etc., are present at the very surface of sample, atop the graphene overlayer, thereby suggesting physisorption (Fig. 3b in the main text). A special case is represented by the OH^- species which appears to be located in two layers, one at the very surface corresponding to the physisorbed water (due to air exposure) and another seemingly chemisorbed in the graphene overlayer (Fig. 3b in the main text).

An oxidized, organic, partial monolayer (~0.4 nm thick), presumably an acetone or, most probably, a PMMA/acetone residue is represented by the C_2O^- marker and can be observed right under the graphene, followed by a third layer containing traces of copper residue (Fig. 3b in the main text). In fact, a closer look at the shape of the C_2N^- depth profile suggests that most of its signal originates from three atomic-like layers, confirming the three layered structure of the transferred graphene system. A thicker, fourth layer, related to the adventitious

chemisorbed organic material at the h-BN surface, has to be taken into account to explain the full C_2N^- profile. Further, assuming a similar ionization probability for nitrogen in each of the first three layers, the fit of the C_2N^- profile with a sum of four Voigt functions convoluting equal shares of Gaussian and Lorentzian functions, with the first one being constrained to monolayer graphene width (Supplementary Fig. 8), leads to a relative quantification of the amount of nitrogen residue in the graphene overlayer, the PMMA/acetone residue underlayer, and the copper-doped third layer, with ratios of about 1 to 0.58 to 0.19, respectively. Consequently, assuming an isotropic distribution of the nitrogen residue in each graphene-related layer, the coverage of these three layers reads 1 ML, 0.58 ML and 0.19 ML, respectively, where the first layer is considered to be, roughly, a full monolayer graphene whereas the second and third a combination of organic residuals consisting of nitriles, carbon sulfides and organic oxides that follow the copper wet etching and PMMA removal procedures. The fourth layer is related to the initial h-BN surface thus not accounted as part of the transferred graphene system.

Finally, the copper density at the graphene/h-BN interface was estimated at ~0.05 % of the bulk copper density (8960 kg m^{-3}) based on direct comparison of Cu^- secondary ion signals between graphene/h-BN and graphene/copper foil systems. Knowing the graphene unit cell (rhombic structure) contains 2 carbon atoms in an area of $\frac{3\sqrt{3}}{2}a^2 \cong 0.0524$ nm^2, where $a = 0.142$ nm is the honeycomb lateral size, the graphene surface density reads about 0.76 mg m^{-2}. As a result, considering its 3 layer structure (1 ML, 0.58 ML and 0.19 ML) extending over a depth of about 1.2 nm, the transferred graphene system contains ~0.4% copper of its total mass (or less than 0.08 atomic %). However, given its depth localization, the copper residue appears to be spatially decoupled from the graphene overlayer thus assumed not to contribute to its defect density. Moreover, it is virtually undetectable by other spectroscopic techniques, e.g. x-ray photoelectron spectroscopy (XPS), accounting for less than 0.03% of the mass (or less than 0.006 atomic %) in the first 10 nm of the graphene/h-BN surface. This value is calculated by estimating the number of atomic layers in the first 10 nm of the graphene/h-BN system at about 30, 3 of which originating from the transferred graphene system and 27 from the h-BN substrate, and by knowing the h-BN density, ~2100 kg m^{-3}.

Supplementary Note 6. Surface localization of residues

Supplementary Fig. 9 presents a series of secondary ion maps (50 x 50 μm^2) recorded in high lateral resolution (~200 nm) mode with a Bi_3^+ analysis ion beam. These maps are recorded after ~0.3 nm of the surface has been removed by Cs^+ sputtering at 500 V energy and represent

the main species of interest related to the graphene overlayer (C_3^-), chemisorbed copper solvent residues (C_2N^-), chemisorbed water (OH^-), PMMA/acetone residues partial underlayer (C_2O^-), chemisorbed adventitious organic material at the h-BN flake surface (CB^-), and the SiO_2 substrate (SiO_2^-). Due to the intrinsically very low current of the analysis ion beam when using bursting in high lateral resolution mode, Bi_3^+ clusters were preferred instead of Bi_1^+ as analysis ion beam species knowing that polyatomic sputtering increases the secondary ion yield of organic fragments (C_3^-, for example)[8]. In this case, bursting was needed to add the high mass resolution capability otherwise unavailable in the high lateral resolution mode (see Fig. 2a and 2b in the main text). Large defect areas are visible in the C_3^- maps corresponding to graphene patches inherent to the transfer process. Having the same in-plane localization, the C_3^-, C_2N^-, S^- (Supplementary Fig. 9), and C_2O^- secondary ion signals indicate that the ammonia persulfate and PMMA/acetone residues are uniformly distributed, at ranges >200 nm (as far as the TOF-SIMS lateral resolution permits), within the three-layered graphene system (i.e. they are chemisorbed). In addition, as the depth profiles of C_2N^- and S^- extend over the depth profiles of C_3^- and C_2O^- (Supplementary Fig. 7), we conclude that cyanide and sulfide compounds are most probably chemisorbed in the graphene layer during the copper wet etching process and further expand into two partial adlayers, leading to a passivation effect. Finally, upon PMMA removal, the organic oxide residues most probably diffuse through the graphene overlayer or its grain boundaries and stabilize right below in the second adlayer. The CB^- and SiO_2^- maps show the position of the h-BN flake and the graphene grain boundaries (i.e., exposed substrate), respectively. As a clear indication of being a PMMA/acetone marker, the C_2O^- map follows both the C_3^- and SiO_2^- maps, as expected for a solvent that was used after the deposition of the PMMA/graphene system onto the h-BN/SiO_2 substrate. Given that the physisorbed species (~0.3 nm) were previously removed by sputtering, the secondary ion map of the OH^- species represents the chemisorbed water which appears uniformly distributed within the plane of the graphene overlayer and exposed SiO_2 substrate. Additional contaminants were below the detection limit or their signal was too low to produce a reasonable image, as in the case of Cu^-. Albeit impossible to spatially map in plane, the copper residue is most probably isotropically chemisorbed at the bottom of the graphene overlayer in the lower ammonia persulfate residual layer, as inferred in Fig. 3b in the main text, following the wet etching process of the initial copper foil support.

Supplementary Note 7. Application to other 2D heterostructures: graphene-on-MoS_2

The technique described here can be broadly applied to other 2D heterostructures. To capture the many excellent and differing material properties of 2D materials, the heterostructures will become increasingly complex. In order to demonstrate the broad applicability of this method to 2D heterostructures, a sample was prepared with CVD MoS_2 and CVD graphene. The MoS_2 was prepared via CVD with MoO_3 and S by a previously reported process[9]. The CVD graphene was produced with the same method as the graphene and h-BN heterostructure that is discussed in depth in this paper. The graphene was transferred onto the $MoS_2/SiO_2/Si$ substrate with the same previously discussed process as well. This 2D heterostructure was also analyzed with TOF SIMS in the high resolution chemical mapping (BA) mode. Supplementary Fig. 10 shows the false-color overlaid maps of C_2^-, S^-, and O^- secondary ions (with colors red, green and blue, respectively) after 0-4 s (Supplementary Fig. 10a) and 50-54 s (Supplementary Fig. 10b) of Cs^+ sputtering.

Supplementary References

1. Zimmerman, J. D. *et al.* Control of interface order by inverse quasi-epitaxial growth of squaraine/fullerene thin film photovoltaics. *ACS Nano* **7,** 9268–75 (2013).

2. Hofmann, S. Sputter depth profile analysis of interfaces. *Reports Prog. Phys.* **61,** 827–888 (1998).

3. Hofmann, S. Profile reconstruction in sputter depth profiling. *Thin Solid Films* **398-399,** 336–342 (2001); Hofmann, S. From Depth Resolution to Depth Resolution Function: Refinement of the Concept for Delta Layers, Single Layers and Multilayers. *Surf. Interface Anal.* **27**, 825–834 (1999).

4. Elko-Hansen, T., Dolocan, A. & Ekerdt, J. G. Atomic Interdiffusion and Diffusive Stabilization of Cobalt by Copper During Atomic Layer Deposition from Bis (N-tert-butyl-N'-ethylpropionamidinato) Cobalt (II). *J. Phys. Chem. Lett.* **5,** 1091–1095 (2014).

5. Sai, N. *et al.* Understanding the Interface Dipole of Copper Phthalocyanine (CuPc)/C60: Theory and Experiment. *J. Phys. Chem. Lett.* **3,** 2173–2177 (2012).

6. Berglund, S. P. *et al.* p-Si/W2C and p-Si/W2C/Pt photocathodes for the hydrogen evolution reaction. *J. Am. Chem. Soc.* **136,** 1535–44 (2014).

7. Ferrari, A. C. & Basko, D. M. Raman spectroscopy as a versatile tool for studying the properties of graphene. *Nat. Nanotechnol.* **8,** 235–46 (2013).

8. Ngo, K. Q. *et al.* Analysis and fragmentation of organic samples by (low-energy) dynamic SIMS. *Surf. Interface Anal.* **43,** 88–91 (2011).

9. Van der Zande, A. M. *et al.* Grains and grain boundaries in highly crystalline monolayer molybdenum disulphide. *Nat. Mater.* **12,** 554–61 (2013).
